# OXA-181-producing *Klebsiella pneumoniae* establishing in Singapore

**DOI:** 10.1186/1471-2334-13-58

**Published:** 2013-02-01

**Authors:** Michelle N D Balm, Grace Ngan, Roland Jureen, Raymond T P Lin, Jeanette W P Teo

**Affiliations:** 1 Department of Laboratory Medicine, National University Health System, 5 Lower Kent Ridge Road, Singapore, 119074, Singapore

**Keywords:** Gram negative resistance, Carbapenemases, Hospital associated infections, Beta-lactamases

## Abstract

**Background:**

Carbapenemase producing *Enterobacteriaceae* are becoming a major public health concern globally, however, relatively little is known about the molecular and clinical epidemiology of these organisms in many parts of the world.

**Methods:**

As part of a laboratory surveillance programme, 96 carbapenem non-susceptible Enterobacteriaceae isolates from clinical samples from patients in seven hospitals were referred for investigation for carbapenemases. Using polymerase chain reaction (PCR) to screen for a collection of genes encoding carbapenemases, 33 of 96 (34.5%) isolates were confirmed as carbapenemase producers. NDM-1 producers were the most prevalent at 64% (21/33) whilst OXA-181 was the second most common carbapenemase constituting 24.5% (8/33) of the carbapenemase producing isolates. Seven of these eight OXA-181 positive isolates underwent further characterisation with screening for other transmissible antimicrobial resistance determinants using PCR. Clonal relatedness was explored using Multilocus sequence typing (MLST) and Pulsed Field Gel Electrophoresis (PFGE). Plasmid characterisation was performed including restriction analysis and transfer by conjugation or transformation.

**Results:**

In addition to the OXA-181 gene, all contained other transmissible resistance determinants including extended spectrum β-lactamases, oxacillinases or 16S rRNA methylase genes, but none contained metallo-β-lactamases or serine carbapenemases. All isolates had a multidrug resistant phenotype with two isolates being resistant to every antibiotic tested including colistin. Multilocus sequence typing confirmed five isolates belonged to ST17 and two to ST14, with those belonging to the same sequence type having identical PFGE profiles. The OXA-181 gene was typically carried on large plasmids which were mostly non-conjugative.

**Conclusions:**

OXA-181 carbapenemase appears to be an important and probably under-recognised cause of carbapenem resistance in Enterobacteriaceae in Singapore. Further coordinated research into clinical and molecular epidemiology of carbapenemases is urgently required in Singapore and throughout Asia.

## Background

Acquired resistance to carbapenems in *Enterobacteriaceae* is a major concern worldwide. This is due both to their importance as human pathogens especially within the hospital setting, and to the highly transmissible nature and propensity for rapid spread shown by these organisms. An increasingly diverse range of enzymes are being recognised as significant, including serine proteases of Ambler Class A, the Class B metallo-β-lactamases and carbapenem-hydrolysing Class D oxacillinases [1]. Originally found in *Shewanella* species, OXA-48-like carbapenemases have now emerged in *Enterobacteriaceae*, predominantly *Klebsiella pneumoniae*. These carbapenem hydrolysing Class D β-lactamases are unusual among carbapenemases in their weak hydrolysis of cephalosporins and resistance to inhibitors such as clavulanate, making them difficult to identify in the laboratory [2]. OXA-181, a variant of OXA-48, was initially reported in India but has been sporadically detected in the United Kingdom, The Netherlands, France, New Zealand, Oman and Singapore [2-7]. Molecular characterisation of this emerging variant remains limited.

Until recently, the vast majority of carbapenem resistance in this group of organisms was due to extended spectrum β-lactamase (ESBL) or AmpC cephalosporinase production combined with impermeability due to porin mutations [8]. Carbapenemase producing organisms were infrequently reported. Between October 2010 and March 2012, we screened 96 *Enterobacteriaceae* clinical isolates with MIC ≥ 2 mg/L to meropenem or imipenem for acquired carbapenemases using polymerase chain reaction (PCR). Isolates were referred for investigation from seven of eight (87.5%) hospital or community laboratories in Singapore after initial antimicrobial susceptibility testing showed non-susceptibility to carbapenems. Epidemiological data was not provided by referring laboratories. Carbapenemase genes were detected in 33 (33/96, 34.4%), including eight clinical isolates of OXA-181-producing *Klebsiella pneumoniae* from patients in three different hospitals. NDM-1 producing isolates dominated this study at 64% (21/33) whilst OXA-181 producers constituted 24.5% (8/33) of carbapenemase producing isolates thus making *bla*_OXA-181_ the second most detected carbapenemase gene after *bla*_NDM-1_[9]. The remaining were KPC producers (4/33) [10]. Here, we report the molecular characterisation of seven out of the eight of the OXA-181 producing isolates. One isolate was excluded from analysis as consent was not obtained from the referring laboratory.

## Methods

All isolates were confirmed as *K*. *pneumoniae* using matrix assisted laser desorption ionisation-time of flight-mass spectrometry (MALDI-ToF-MS, Bruker Daltonics GmHB, Bremen, Germany). Antimicrobial susceptibility testing was performed with VITEK-2 instrument and carbapenem MICs confirmed with Etest (bioMérieux, Marcy L’Etoile, France) with susceptibility defined according to European Committee on Antimicrobial Susceptibility Testing (EUCAST) breakpoints. Presence of carbapenemases were screened phenotypically using disc diffusion assays with meropenem discs supplemented with boronic acid, cloxacillin or dipicolinic acid (Rosco Diagnostica A/S, Taastrup, Denmark) and the modified Hodge test [11].

Isolates underwent screening for transmissible β-lactamase genes including serine carbapenemases (KPC-type), metallo-β-lactamases (MBL; NDM-type, VIM-type, IMP-type), oxacillinases and extended spectrum β-lactamases (ESBL; TEM-type, SHV-type, CTX-M-type) with PCR followed by sequencing of amplicons [9,10]. Detection of *bla*_OXA_ was based on a multiplex PCR assay developed by Woodford *et al*. [12] with addition of *bla*_OXA-48-like_ primers (OXA-48L 5^′^- GTGGGATGGACAGACGCG-3^′^ and OXA-48 LL 5^′^-CCACACATTATCATCAAGTTC-3^′^) using National Collection of Type Cultures (NTCT) 13442 *K*. *pneumoniae* as positive control for *bla*_OXA-48_. Plasmid mediated 16S rRNA methylase aminoglycoside resistance determinants (*arm*A, *rmt*A, *rmt*B, *rmt*C, *rmtD* and *npm*A) were also analysed by PCR.

Clonal relatedness was investigated using Multilocus sequence typing (MLST, http://www.pasteur.fr/recherche/genopole/PF8/mlst) and Pulsed Field Gel Electrophoresis (PFGE) of SpeI (New England Biolabs, Ipswich, MA) digested genomic DNA [13].

The number and size of plasmids were analysed by S1 nuclease digestion of whole genomic DNA followed by PFGE (S1-PFGE) [14]. In addition, smaller plasmids were extracted using the QIAprep spin miniprep kit (Qiagen GmbH, Hilden, Germany). Southern hybridisation using a digoxigenin (DIG) labelled *bla*_OXA-181_ probe (DIG DNA Labelling and Detection kit, Roche Diagnostics, Mannheim, Germany) was used to localise *bla*_OXA-181_. Transformation studies were performed with plasmid DNA extracted using QIAprep Spin Miniprep kit introduced by electroporation into *E*. *coli* DH5α cells using Gene Pulser Xcell (Bio-Rad, Hercules, CA) with transformants selected on Luria-Bertani (LB) agar supplemented with imipenem (1 mg/L). Conjugation experiments were performed between *K*. *pneumoniae* isolates and azide-resistant recipient *E*.*coli* J53 with transconjugants selected on LB agar containing sodium azide (50 mg/L) and imipenem (1 mg/L). PCR-based replicon typing was used to identify plasmid incompatibility groups [15]. To analyse the immediate genetic environment of the *bla*_OXA-181_ gene in our isolates, primers (IRF 5^′^-CCTAGATTCTACGTCAGTAC-3^′^ and IRR2 5^′^-CTCTCTAGTCGGACAACACC-3^′^) based on GenBank reference sequence NC_019160.1, designed to walk in from the left and right inverted long repeats (IRL) in the direction of *bla*_OXA-181_ were used.

## Results and discussion

As part of a surveillance program for carbapenem non-susceptible Enterobacteriaceae in Singapore, 96 isolates from seven hospitals or private laboratories were submitted for investigation. Of these, 33 clinical isolates contained carbapenemase genes as detected by PCR with eight isolates bearing *bla*_OXA-181_. Consent from referring laboratories was obtained for seven of these isolates to be subjected to molecular characterisation. The *bla*_OXA-181_ positive clinical isolates were confirmed as *Klebsiella pneumoniae* by MALDI-ToF MS. Most showed high level resistance to carbapenems, and interestingly also to cephalosporins (Table 1), suggesting the presence of other resistance determinants in addition to *bla*_OXA-181_. Two of the seven isolates were resistant to all antimicrobial agents tested including colistin. All seven OXA-181 producers were positive by the modified Hodge test. No synergy was detected using disc diffusion assays, the usual pattern seen with oxacillinase presence.

**Table 1:** **Characteristics of OXA-181-producing *Klebsiella pneumoniae *clinical isolates**

**Isolate**	**Hospital**	**Specimen**	**Date of Isolation**	**ST**	**16S rRNA methylase gene**	**β-lactamases**	**Plasmids**		**MIC** (**mg**/**L**)											
							**Size** (**kb**)	**Replicon**(**s**)	**IMP**	**MEM**	**ETP**	**FOX**	**CTX**	**CAZ**	**AZT**	**COL**	**TGC**	**LEV**	**GEN**	**AMK**
KPO8	Hospital A	Tracheal aspirate	18/01/2011	17	*armA*	TEM-116, SHV-11, CTX-M-15, OXA-1	~97, ~170	A/C	>32	>32	>32	>256	>256	64	>256	0.125	2	0.25	>256	>256
KPO9	Hospital A	Sputum	8/02/2011	17	*armA*	TEM-1, SHV-11, CTX-M-15, OXA-1	~9, ~150, ~170	A/C	>32	>32	>32	>256	>256	64	>256	16	>256	>256	>256	>256
KPO7	Hospital A	Tracheal aspirate	9/12/2010	14	*armA*	TEM-1, SHV-1, CTX-M-15, OXA-1, OXA-9	~1.5, ~50, ~170, ~194	A/C, F	>32	>32	>32	>256	>256	>256	>256	0.25	12	>256	>256	>256
KPO26	Hospital A	Sputum	14/05/2011	14	ND	TEM-1, SHV-1, CTX-M-15, OXA-1, OXA-9	~1.6, ~194	A/C	>32	>32	>32	>256	>256	>256	>256	32	4	>256	>256	96
KPO6916	Hospital A	Blood	2/03/2012	14	*armA*	TEM-1, SHV-1, CTX-M-15, OXA-1, OXA-9	~1.6, ~50, ~170, ~194	A/C	8	8	>32	>256	>256	>256	>256	0.25	16	>256	>256	>256
KPO7431	Hospital B	Urine	22/02/2012	14	*armA*	TEM-1, SHV-11, CTX-M-15, OXA-1, OXA-9	~1.5, ~50, ~170, ~194	A/C, F	4	4	>32	>256	>256	>256	>256	0.25	8	>256	>256	>256
KPO83	Hospital C	Urine	30/12/2011	14	*armA*	TEM-1, SHV-1, CTX-M-15, OXA-1, OXA-9	~170	A/C	32	8	>32	>256	>256	>256	>256	0.5	16	32	>256	>256
KPO9–T	―	―	―	―	*armA*	OXA-181, CTX-M-15	~170	A/C	2	0.5	0.5	2	3	1	0.75	0.125	0.125	0.002	>256	>256
KPO26 –T	―	―	―	―	ND	OXA-181, OXA-9	~194	A/C	2	0.19	0.25	12	6	2	6	0.125	0.125	0.003	0.125	1
*E*. *coli* J53	―	―	―	―	―	―	―	―	0.125	0.016	0.04	1	0.064	0.25	0.064	0.125	0.064	0.004	0.125	0.125

Legend:

MIC, minimum inhibitory concentration; IPM, imipenem; MEM, meropenem; ETP, ertapenem; FOX, cefoxitin; CTX, cefotaxime; CAZ, ceftazidime; AZT, aztreonam; COL, colistin; TCG, tigecycline; LEV, levofloxacin; GEN, gentamicin; AMK, amikacin; KPO8, KPO9, KPO7, KPO26, KPO6916, KPO7431, KPO83 carbapenem-resistant *K*. *pneumoniae* clinical isolate; KP09-T, KP026-T, *K*. *pneumoniae* transconjugant from KP09, KP026; J53, *E*. *coli* recipient strain.

Full gene sequencing of amplicons revealed 100% identity with *bla*_OXA-181_ [GenBank: JN205800.1]. PCR screening and sequencing showed that all isolates also carried ESBL genes and at least one additional *bla*_OXA-type_ but none were positive for MLBs or KPC-type β-lactamases (Table 1). *arm*A was detected in six of the seven isolates (Table 1). MLST revealed five isolates of sequence type (ST)14 and two of ST17. Furthermore, isolates belonging to the same sequence type had identical PFGE patterns. OXA-181-producing isolates came from three different hospitals over a 15 month period, and to our knowledge patients did not have an epidemiological link to each other although full data is not accessible.

Analysis of plasmid content using miniprep kit extractions and S1-PFGE revealed the isolates contained multiple small (~1.5kb) and large plasmids (~50 - ~194kb) (Table 1). Southern hybridization studies using a *bla*_OXA-181_ probe localised the gene to a large plasmid of ~170kb in six of the seven clinical isolates and to a ~194kb plasmid in isolate KPO26 (Table 1). Transformation studies were not successful in selecting transformants; however, the method would have only extracted small plasmids. This would support localisation of *bla*_OXA-181_ to large plasmids. Previously *bla*_OXA-181_ has been described in a range of plasmid backbones ranging from a small ColE2-type plasmid of ~7.6kb in *K*. *pneumoniae* KP3 to large plasmids of 200-250kb [3,16]. Transconjugants were obtained for only two isolates (KPO-9, KPO-26) with transfer of a large plasmid carrying *bla*_OXA-181_ demonstrated by S1-PFGE and Southern hybridization analysis (data not shown). Transconjugant KPO-26 remained susceptible to non-β-lactam antibiotics whilst for KPO-9, *armA* was co-transferred with the plasmid (Table 1).

To further characterize the plasmids, PCR- based replicon typing was performed. IncA/C replicons were detected in all isolates including the transconjugants (Table 1), suggesting *bla*_OXA-181_ was present on an IncA/C plasmid although Southern hybridisation analysis using IncA/C probes would be needed to confirm this. Plasmids belonging to the IncA/C incompatibility group are of interest as they carry resistance to diverse groups of antimicrobial agents and have a broad host range [17].

*bla*_OXA-181_ has been found as part of transposon, Tn*2013*, with upstream insertion sequence IS*Ecp1* mediating one-ended transposition of the *bla*_OXA-181_ gene [16]. The immediate genetic environment surrounding *bla*_OXA-181_ of our isolates was investigated. Gene mapping demonstrated presence of the IS*Ecp1* element upstream, however, no PCR products were obtained when attempting to amplify downstream sequences (*Δlys* and *Δere*). This raises the possibility that *bla*_OXA-181_ in our isolates could be present in a structure different to the previously characterized Tn*2013*[16].

The surveillance project from which these isolates were derived is ongoing. This project has previously identified that NDM-1 containing organisms appear to be well established in different *Enterobacteriaceae* species, often in patients without travel history within the preceding year and that KPC-2-producing *Klebsiella pneumoniae* has been introduced to Singapore probably from mainland China [9,10]. Our work suggests that *bla*_OXA-181_ may already have established on plasmids within successful *K*. *pneumoniae* strains in Singapore. There is also the likelihood that a much wider reservoir of carbapenemase-producing *Enterobacteriaceae* already exists here given that this is a voluntary programme and only clinical isolates with obviously elevated carbapenem MICs were referred for investigation. This could be especially true for organisms carrying OXA-48-like carbapenemases which may not exhibit high level resistance to carbapenems or cephalosporins in the absence of other resistance determinants capable of conferring carbapenem non-susceptibility. A major limitation of our study is that clinical data on patients from whom the isolates were obtained is lacking. Although all isolates studied in this collection came from clinical samples, no data is available on whether these represent colonisation or true infection, nor regarding treatment and outcomes. This makes establishing the context of carbapenem resistance in *Enterobacteriaceae* in Singapore more difficult. Indeed, any data on carbapenemase-producing *Enterobacteriaceae* is very limited in South East Asia. Given the frequency of travel and economic migration through many parts of the region and the widespread resistance to other antimicrobial classes, carbapenemase production in *Enterobacteriaceae* is likely to be an under-estimated entity, made more difficult by the lack of availability for diagnostic testing in many areas.

## Conclusion

OXA-181 carbapenemase appears to be an emerging and probably under-recognised cause of carbapenem resistance in Enterobacteriaceae in Singapore. This preliminary data, along with the work of others [7], has provided the basis for establishing further coordinated clinical and molecular epidemiological research into carbapenemase-producing Enterobacteriaceae in Singapore aimed at addressing many of the gaps in our current understanding.

## Abbreviations

ESBL: Extended spectrum β-lactamase; KPC: Klebsiella pneumoniae carbapenemase; MALDI-ToF-MS: Matrix assisted laser desorption ionisation-time of flight-mass spectrometry; MBL: Metallo-β-lactamase; MIC: Minimum inhibitory concentration; MLST: Multilocus sequence typing; NDM: New Delhi metallo-β-lactamase; OXA: Oxacillinase; PCR: Polymerase chain reaction; PFGE: Pulsed field gel electrophoresis; ST: Sequence type.

## Competing interests

The authors declare that they have no competing interests.

## Authors’ contributions

MB conceived and drafted the study design, contributed to analysis of the data and wrote the primary draft of the manuscript. GN performed the laboratory experiments. RJ and RL participated in the study design and helped to draft the manuscript. JT conceived and drafted the study design, performed the laboratory experiments and data analysis and helped to draft the manuscript. All authors read and approved the final manuscript.

## Pre-publication history

The pre-publication history for this paper can be accessed here:

http://www.biomedcentral.com/1471-2334/13/58/prepub
